# Effects of virtual reality motor games on motor skills in children with cerebral palsy: a systematic review and meta-analysis

**DOI:** 10.3389/fpsyg.2024.1483370

**Published:** 2025-01-06

**Authors:** Zhuolin Xue, Weiqiang Zhang, Ni Zhou, Pengwei Ma, Kun Yuan, Peiyun Zheng, Junfeng Li, Jindong Chang

**Affiliations:** ^1^School of Physical Education, Southwest University, Chongqing, China; ^2^Institute of Motor Quotient, School of Physical Education, Southwest University, Chongqing, China; ^3^Department of Physical Education, Central South University, Changsha, China; ^4^Ministry of Sports, Shandong Technology and Business University, Yantai, China

**Keywords:** virtual reality, cerebral palsy, gross motor skills, fine motor skills, children

## Abstract

**Background:**

Enhancing motor skills is crucial for the functional development of children with cerebral palsy. Virtual reality has emerged as a promising technology for rehabilitating these children.

**Objective:**

The objective of this study was to systematically evaluate the effects of virtual reality motor games on the gross and fine motor skills of children with cerebral palsy.

**Methods:**

A comprehensive search was conducted in databases including PubMed, ProQuest, EBSCOhost, Web of Science, and Wanfang Data, covering publications from their inception to June 1, 2024, to identify randomized controlled trials (RCTs) investigating the effects of virtual reality motor games on the gross and fine motor skills of children with cerebral palsy. The quality of the included studies was assessed using the PEDro scale, and data were analyzed with RevMan software (version 5.4).

**Results:**

Nineteen studies involving 850 children with cerebral palsy were included. The results indicated that virtual reality motor games significantly improved gross motor skills [mean difference (MD) 1.67, 95% confidence interval (CI): 0.75–2.56; *P* < 0.001] and fine motor skills [standardized mean difference (SMD) 0.73, 95% CI: 0.30–1.16; *P* = 0.00008] in children with cerebral palsy.

**Conclusions:**

Virtual reality motor games have the potential to significantly enhance both gross and fine motor skills in children with cerebral palsy.

**Systematic review registration:**

https://www.crd.york.ac.uk/prospero/, PROSPERO [CRD42024558713].

## 1 Introduction

Cerebral palsy (CP) is a collection of permanent motor and postural developmental disorder, characterized by limited movement resulting from non-progressive brain abnormalities occurring during fetal or early infant development (Colver et al., 2014). As the most prevalent physical disability in childhood, CP affects approximately 1 in 500 live births (Novak et al., 2017). Typically, CP is diagnosed clinically before the age of two, based on a combination of symptoms and neurological signs such as spasticity, dyskinesia, ataxia, hyperreflexia, and, in rare cases, hypotonia (Brandenburg et al., 2019). Around 6–15% of children with CP experience involuntary movements, including athetosis and chorea, which are marked by changes in muscle tone and involuntary movements. Ataxic CP primarily presents with balance and coordination difficulties, potentially accompanied by involuntary movements and tremors of the limbs. Hypotonia, or hypotonic CP, is a rare occurrence, found in only 2% of cases, and is characterized by excessively floppy muscles (Twum and Hayford, 2004). Furthermore, over 80% of children with CP exhibit spasticity (Arneson et al., 2009). Despite being considered a non-progressive disease, the movement impairments associated with CP often become more pronounced as children grow (Jiang et al., 2024).

The development of motor skills in individuals with CP is diverse, with some children demonstrating significant progress while others continue to face challenges. Gross motor skills, which involve the use of large muscle groups for position changes and balance maintenance, and fine motor skills, which include movements of the fingers, hands, and wrists for tasks such as object manipulation, writing, and dressing, are typically less developed in children with CP compared to their normally developing peers. This is evident in various aspects of motor skill development, including strength, balance, agility, flexibility, coordination (particularly hand-eye coordination), proprioception, and reaction time. These movement disorders not only impede daily activities but can also lead to secondary musculoskeletal issues, such as hip dislocation and impaired hand function. Therefore, enhancing the movement capabilities of children with CP is crucial for improving their quality of life.

The motor rehabilitation of children with CP has garnered significant attention from a multitude of departments and governmental agencies both domestically and internationally. Among the myriads of rehabilitation technologies available, virtual reality (VR) technology distinguishes itself due to its interactive nature, patient-centric methodology, and cost-efficiency. VR constitutes an interactive computer simulation system grounded in a three-dimensional environment. This system predominantly offers users an immersive experience via auditory and visual feedback, with occasional incorporation of tactile feedback (Gorini and Riva, 2008). VR technology has gained significant traction in the field of neurorehabilitation, owing to its engaging, interactive, patient-centric, and relatively cost-effective attributes (Bargeri et al., 2023). In the context of rehabilitation, VR actively promotes the involvement of children with CP by stimulating their visual, auditory, tactile, and kinesthetic senses. This stimulation synchronizes central neural conduction with peripheral motor control, thereby contributing to their rehabilitation progress (Liu et al., 2022). Traditional rehabilitation training methods are often monotonous, leading to decreased motivation and participation, which in turn can negatively impact the effectiveness of the rehabilitation process. In contrast, highly interactive and immersive VR exercise games have been shown to enhance treatment compliance and increase the frequency of repeated movements (Zhang et al., 2024). Consequently, individuals who might be disinclined to engage in conventional CP treatments for children may prefer VR-based rehabilitation (Harris and Reid, 2005). These VR exercise games not only bolster the gross motor functions and daily living skills of children with cerebral palsy but also mitigate spasticity and elevate their quality of life. Studies indicate that VR exercise games are particularly effective in enhancing upper limb function and hand mobility, especially when used as an adjunct to traditional treatments (Soleimani et al., 2024).

The application of virtual reality (VR) technology in rehabilitation is increasingly prevalent, prompting researchers to examine its short-term and long-term impacts on the motor skills of children with cerebral palsy (CP). Studies suggest that VR rehabilitation training positively influences the upper limb motor functions of children with hemiplegic cerebral palsy. Sajan et al. (2017) conducted a study involving virtual rehabilitation training on ten children with cerebral palsy, finding an improvement in hand coordination post-training. Similarly, Juan et al. (2023) demonstrated that virtual reality rehabilitation effectively enhances upper limb functional motor skills and movement quality in children with spastic hemiplegia and cerebral palsy. These findings indicate that VR technology can rapidly improve the motor skills of children with cerebral palsy. Regarding gait training, a meta-analysis by Han Jing et al. revealed that VR rehabilitation training, when combined with conventional rehabilitation training, can significantly facilitate the recovery of lower limb function in children with cerebral palsy.

Winter et al. (2021) implemented an immersive virtual reality walking training program for 16 children aged 7–16 years, who were diagnosed with cerebral palsy. The training was conducted over a period of 4 weeks, with a frequency of 5 times per week and a duration of 30 min per session. The results indicated a significant improvement in the gross motor function test scores in area D (standing) and area E (walking, running, jumping) of the table (GMFM), compared to the baseline period. Additionally, there was a notable enhancement in walking speed, as well as hip and ankle joint mobility. These findings not only validate the short-term effectiveness of VR technology, but also suggest that its benefits are relatively enduring. Consequently, these results underscore the potential of VR technology in the rehabilitation of children with cerebral palsy, particularly in terms of enhancing motor skills and fostering long-term functional improvements (Winter et al., 2021).

Previous meta-analyses have demonstrated that VR technology can substantially improve children's gross motor function and fine motor skills (Liu et al., 2022; Ren and Wu, 2019; Abdelhaleem et al., 2022; Bell et al., 2024; Montoro-Cárdenas et al., 2022). The primary objectives of this study were to address the following questions: (1) Can the introduction of new evidence enhance the assessment of the effectiveness of VR motor games in improving motor skills in children with CP? (2) Is there a difference in the improvement of motor skills in children with CP based on different frequencies and durations of VR motor game training? (3) Do the results measured using different assessment scales affect the study's conclusions? Addressing these questions will provide a scientific basis for developing an effective VR motor game training program for children with CP.

## 2 Methods

This study adhered to international guidelines for conducting meta-analyses, specifically following the PRISMA (Preferred Reporting Items for Systematic Reviews and Meta-Analyses) statement, which provides a framework for selecting and applying methods in evaluating healthcare interventions. The registration number was CRD42024558713 in the PROSPERO.

### 2.1 Search strategy

Two independent researchers conducted a comprehensive search of five databases: PubMed, ProQuest, EBSCOhost, Web of Science, and Wanfang Data. The objective was to identify randomized controlled trials (RCTs) that assessed the effects of virtual reality motor games on motor skills in children with cerebral palsy. This search encompassed the timeframe from the inception of each database up until June 1, 2024. In addition to this primary search, relevant systematic reviews and citations from the selected studies were also considered as supplementary sources. The search strategy incorporated both subject-specific terms and free-text keywords. Boolean operators such as “AND” and “OR” were used to effectively combine and sequence these terms, following iterative prechecks. Key search terms encompassed “virtual reality game,” “exergame,” “active video game,” “PlayStation,” “video game,” “Wii,” “Kinect,” “children,” “cerebral palsy,” “little disease,” “infantile palsy,” “spastic diplegia,” “motor skill,” “gross motor skill,” “fine motor skill,” and “training upper extremity function.”

### 2.2 Inclusion criteria

The inclusion criteria were as follows:

(1) Participants: Children with a clinical diagnosis of spastic CP, up to 18 years of age; no restrictions were placed on race or gender.

(2) Intervention: VR motor games or VR motor games combined with rehabilitation training.

(3) Comparison: Daily physical activity, standard or integrated rehabilitation training.

(4) Outcome: Fine motor skills were assessed using the Peabody Developmental Motor Scales-Second Edition (PDMS-2), the Manual Ability Classification System (MACS), the Multidimensional Assessment of Interoceptive Awareness Version 2- Chinese (MA-2), the Box and Block Test (BBT), the Quality of Upper Extremity Skills Test (QUEST), the Minnesota Manual Dexterity Test (MMDT), and the Nine-Hole Peg Test (NHPT). Gross motor skills were evaluated using the Gross Motor Function Measure (GMFM), including the GMFM-66 and GMFM-88, and the Movement Assessment Battery for Children - Second Edition (MABC-2).

(5) Study design: Randomized controlled trials only. The study design exclusively involved randomized controlled trials (RCTs) as the research type. This choice was informed by the unique benefits of RCTs in assessing intervention effects. Randomized controlled trials are widely recognized as the gold standard for evaluating medical interventions, primarily due to their capacity to minimize selection bias and confounding variables. By randomly assigning participants to either experimental or control groups, we ensured that both groups were comparable at baseline. This increases the probability that any observed difference in effect is attributable to the intervention itself, rather than other uncontrolled factors.

Considering the importance of early intervention, we selected subjects under 18 years old. The theory of “peak motor skill development” suggests that the age range from 1 to 12 years is a sensitive period for motor skill development, and the entire childhood period before 18 years old is a critical period for the development and evaluation of basic motor skills. Therefore, it is particularly important to intervene and evaluate children in this age range. Interventions and assessments during this time are crucial for promoting long-term motor skill development, physical health, and mental health of children. By selecting this age group and using a strict RCT design, we can obtain the most convincing evidence on the effectiveness of VR motor games and rehabilitation training combined with VR motor games in improving motor skills in children with spastic cerebral palsy.

### 2.3 Exclusion criteria

The exclusion criteria were implemented as follows:

(1) Studies were excluded based on titles and abstracts if they were theoretical studies, systematic reviews, or if the subjects were adults or older adults.

(2) Studies lacking a control group or those using other interventions were excluded.

(3) Articles were excluded if the outcome indicators did not meet the analysis requirements or if the outcome data were missing. In cases where studies were excluded due to insufficient data, efforts were undertaken to contact the respective authors to procure Supplementary material.

### 2.4 Literature screening and data extraction

Two investigators (ZX and NZ) independently extracted the basic information and outcome data for the included studies within 2 months. If there were any discrepancies between their extractions, a third researcher (JC) would discuss with them to reach a consensus. The extracted data included details such as author, publication year, country, age, sample size, and type of CP, content and frequency of intervention, and outcome indicators. The detailed tables were made by carefully reviewing and recording the relevant information in the articles that met the inclusion criteria.

### 2.5 Quality assessment of included studies

Two independent assessors (PM and KY) evaluated the risk of bias in the included studies using the PEDro scale. In disagreements, a third assessor (PZ) facilitated a discussion to reach a consensus. Once consensus was achieved, the assessors decided on the study quality. The evaluation considered the following 11 criteria, each worth one point: (a) eligibility criteria were clearly defined; (b) subjects were randomly allocated to groups; (c) allocation was concealed; (d) groups were similar at baseline for key outcome measures; (e) all subjects were blinded; (f) all therapists were blinded; (g) all evaluators were blinded; (h) outcome data were obtained from more than 85% of the initially assigned subjects; (i) data for critical outcomes were analyzed using intention-to-treat when applicable; (j) statistical comparisons between groups for at least one key outcome were reported; (k) the study provided point estimates and measures of variability for at least one key outcome. The maximum score for each study was 11, with a score of 7 or higher indicating low risk of bias, 5–6 indicating moderate risk, and four or lower indicating high risk.

### 2.6 Data analysis

Cochrane's software (version 5.4) was used, and the PRISMA guidelines were followed. The *Q-*statistical test (*p*-value) and *I*^2^ statistic were used to assess heterogeneity. If there was statistical heterogeneity between studies (*I*^2^ > 50%; *p* < 0.10), a random-effects model was used for meta-analysis; otherwise, a fixed-effects model was used. For gross motor skills, the results of MABC-2, GMFM-66, GMFM-88, and GMFM were combined. The data from PDMS-2, MACS, MA-2, BBT, QUEST, MMDT, and Nine-hole Peg Test were combined for fine motor skills. Therefore, the mean difference (MD) was used to calculate gross motor skills, and the standardized mean difference (SMD) was used to calculate fine motor skills. There were no significant differences in outcome variables between groups at baseline. After the study, effect sizes were calculated by comparing the scale scores of the intervention group with those of the control group. Each effect size is presented as a point estimate with a 95% confidence interval (CI). A significant difference was found between the intervention and control groups when *p* < 0.05, which means that the meta-analysis results were statistically significant.

### 2.7 Tools for assessment

#### 2.7.1 Gross motor skills

(1) Gross Motor Function Measure (GMFM): GMFM is a standardized, observational assessment tool specifically designed for children with CP. It aims to quantify gross motor function over time and includes two versions: GMFM-66 and GMFM-88. These versions evaluate a child's ability to perform a range of motor tasks, including sitting, crawling, standing, and walking. The GMFM is internationally recognized as the gold standard for assessing motor disabilities, planning rehabilitation treatments, and evaluating the effectiveness of interventions in children with CP.

(2) Movement Assessment Battery for Children - Second Edition (MABC-2): The MABC-2 is a comprehensive test designed to evaluate motor proficiency in children aged 3–16 years. It assesses a wide range of movement skills, encompassing manual dexterity, aiming and catching, and balance. This tool is instrumental in identifying children with motor difficulties and tracking changes in their motor skills over time.

#### 2.7.2 Fine motor skills

(1) Peabody Developmental Motor Scales-Second Edition (PDMS-2): The PDMS-2 is a comprehensive assessment of early childhood motor development, providing an in-depth evaluation of both gross and fine motor skills. It consists of six subtests, including reflexes, grasping, locomotion, object manipulation, and visual-motor integration, measuring the interrelated motor abilities that develop early in life.

(2) Manual Ability Classification System (MACS): The MACS is an observational tool used to classify how children with CP use their hands in daily activities. It provides a descriptive classification of manual ability in children with CP, focusing on what the child does in terms of handling objects in everyday contexts.

(3) Multidimensional Assessment of Interoceptive Awareness Version 2-Chinese (MA-2): The MA-2 is a tool for assessing interoceptive awareness, which is the ability to perceive and understand signals from within the body. This tool is relevant for understanding how children with CP perceive their own bodies and their movements.

(4) Box and Block Test (BBT): The BBT is a simple, reliable, and valid test of fine motor function, particularly for assessing manual dexterity and hand function in children with CP.

(5) Quality of Upper Extremity Skills Test (QUEST): The QUEST is a standardized assessment tool that evaluates the quality of movement of the arms and hands in children with disabilities, including those with CP.

(6) Minnesota Manual Dexterity Test (MMDT): The MMDT is a test of manual dexterity that measures the speed and accuracy of fine motor movements, particularly useful for evaluating hand function in children with CP.

(7) Nine-Hole Peg Test (NHPT): The NHPT is a widely used test for assessing fine motor function and hand dexterity, particularly useful for evaluating the effects of interventions on hand function in children with CP.

## 3 Results

### 3.1 Search results

**Figure 1:**
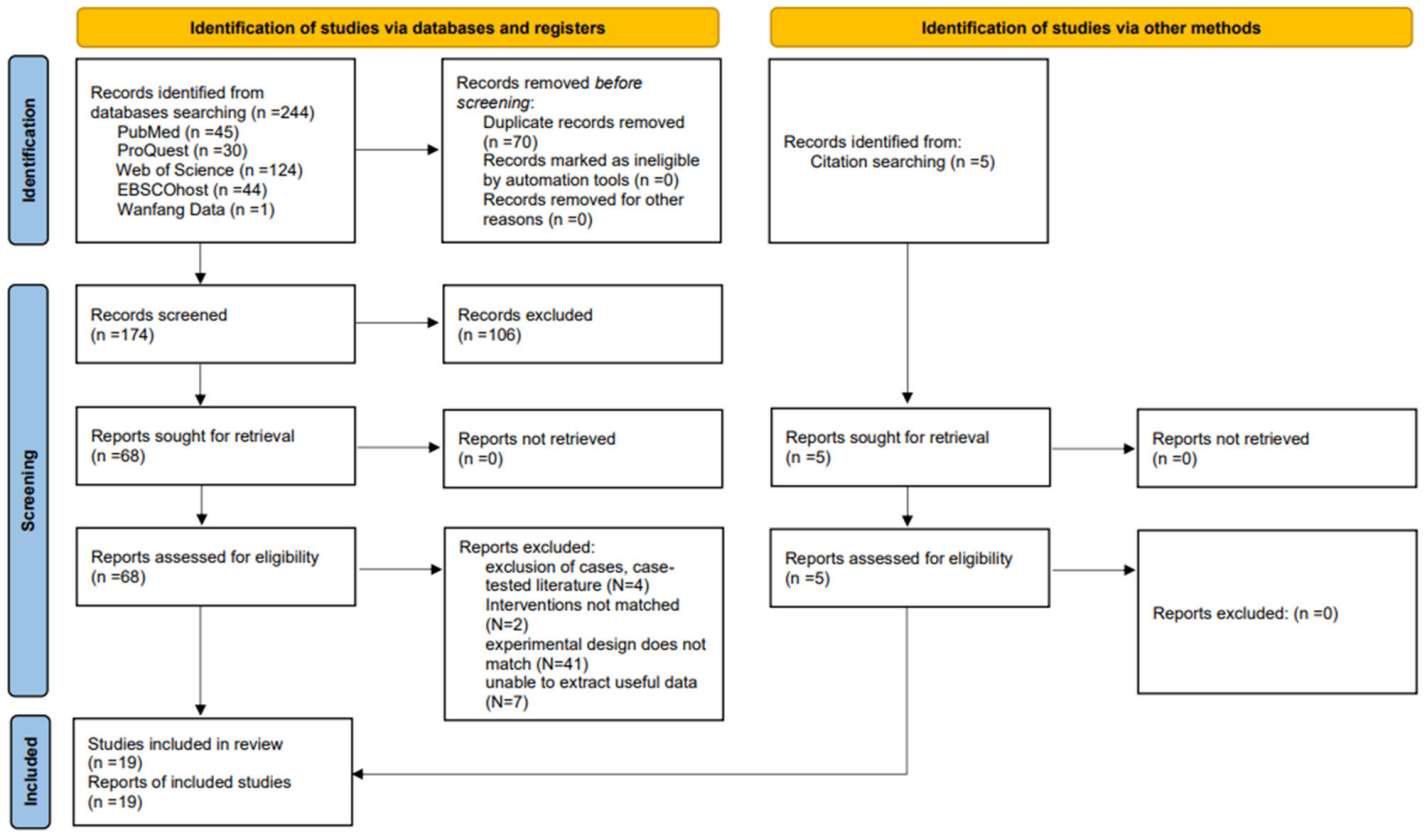
PRISMA flowchart of included and excluded studies.

A total of five databases were searched: PubMed (*n* = 45), ProQuest (*n* = 30), EBSCOhost (*n* = 44), Web of Science (*n* = 124), and Wanfang Database (*n* = 1). After excluding duplicates, 179 articles remained. Following an initial screening, 73 articles were retained; after a further full-text screening, 19 articles were finally included. As a result, 24 RCTs (Decavele et al., 2020; Pin and Butler, 2019; Fidan and Genç, 2023; El-Shamy and El-Banna, 2020; AlSaif and Alsenany, 2015; Bruno Arnoni et al., 2019; Ren et al., 2016; Chang et al., 2020; Chen et al., 2013; Saussez et al., 2023; Acar et al., 2016; Kanitkar et al., 2023; Jha et al., 2021; Cho et al., 2016; Avcil et al., 2021; Chiu et al., 2014; Sharan et al., 2012; Choi et al., 2021; Zhao et al., 2018) were finally included, as shown in Figure 1.

### 3.2 Basic characteristics of included studies

Table 1 shows the basic characteristics of the included studies. Nineteen articles covering 24 RCTs were included, including one article in Chinese and 18 in English. The publication years of the selected literature ranged from 2012 to 2023, and the publication countries included China, Saudi Arabia, Brazil, Canada, Belgium, Turkey, India, and South Korea. A total of 850 children with CP were included in these studies, and the main types of CP included spastic diplegia and spastic hemiplegia. Interventions in the experimental group included VR training and a combination of VR training and regular rehabilitation training; the control group received interventions such as daily physical exercise or regular rehabilitation training. The duration of VR training ranged from 15 to 60 min per session, with a frequency of 2–7 sessions per week and 3 to 12 weeks.

**Table 1:** Basic characteristics of the included studies.

**References**	**Country**	**Age (years)**	**Sample size (E/C)**	**CP type**	**VR intervention**	**Dosage**	**Outcome indicators**
Acar et al. (2016)	Istanbul	Range 6–15	15/15	Spastic Diplegia	Nintendo Wii	15 min/d^*^2 d/wk^*^6 wk	QUEST
Avcil et al. (2021)	Istanbul	Mean:10.0 ± 3.0	15/15	Not specified	Nintendo Wii and LMC Games	60 min/d^*^3 d/wk^*^8 wk	MMDT
Chang et al. (2020)	South Korea	VR:6.08 ± 1.77 CG:4.88 ± 1.15	10/7	Not specified	RAPAEL Smart Kids	20 min/d^*^2 d/wk^*^8 wk	QUEST
Chiu et al. (2014)	China-Taiwan	Range 6–13	32/30	Spastic Diplegia	Wii Sports ResortTM	40 min/d^*^3 d/wk^*^6 wk	Nine-hole Peg Test
Choi et al. (2021)	South Korea	Mean: 5.7 ± 2.8	40/38	Not specified	RAPAEL Smart Kids	60 min/d^*^5 d/wk^*^4 wk	MA-2
El-Shamy and El-Banna (2020)	Saudi Arabia	Range 8–12	20/20	Spastic Diplegia	Nintendo Wii	40 min/d^*^3 d/wk^*^12 wk	PDMS-2
Kanitkar et al. (2023)	Canada	Range 4–10	33/30	Not specified	GRP	45 min/d^*^3 d/wk^*^16 wk	PDMS-2
Saussez et al. (2023)	Belgium	Range 5–18	20/20	Spastic Diplegia	REAtouch	Not specified	BBT
Sharan et al. (2012)	India	VR:8.88 ± 3.23 CG:10.38 ± 4.41	14/15	Not specified	Nintendo Wii fit game	–min/d^*^2 d/wk^*^3 wk	MACS
AlSaif and Alsenany (2015)	Saudi Arabia	Range 6–10	20/20	Spastic Diplegia	Nintendo Wii fit game	20 min/d^*^7 d/wk^*^12 wk	mABC-2
Bruno Arnoni et al. (2019)	Brazilian	Mean: 10 ± 3	7/8	Not specified	Xbox 360 KinectTMand Kinect sensor	45 min/d^*^2 d/wk^*^ 8 wk	GMFM-88
Chen et al. (2013)	China	Range 6–12	14/13	Spastic	Eloton SimCycle	40 mim/d^*^ 3 d/wk^*^ 12 wk	GMFM-66
Cho et al. (2016)	South Korea	VR:10.2 ± 3.4 CG:9.4 ± 3.8	9/9	Spastic	Nintendo Wii jogging program	30 min/d ^*^ 3 d/wk^*^ 8 wk	GMFM
Decavele et al. (2020)	Belgium	Range 6–15	14/13	bilateral spastic	MS Kinect for Windows and Nintendo Wii balance board	45 min/d^*^2 d/wk^*^12 wk	GMFM
Jha et al. (2021)	India	Range 6–12	19/19	bilateral spastic	Kinect-based virtual reality gaming	60 min/d^*^4 d/wk^*^6 wk	GMFM-88
Fidan and Genç (2023)	Istanbul	MeanVR:9.2 MeanCG:9.4	27/25	Spastic	XBox One Kinect	45 min/d^*^2d /8 wk^*^-wk	GMFM-88
Pin and Butler (2019)	China-Hong Kong	EG:8.92 ± 2.25 CG:9.59 ± 1.87	9/9	Not specified	Interactive computer play	20 min/d^*^4 d/wk^*^6 wk	GMFM-66
Ren et al. (2016)	China	Mean 55.3 ± 11.5	19/16	bilateral spastic	Q4 Situational Interactive Training System	40 min/d^*^5 d/wk^*^12 wk	GMFM-88/PDMS-2
Zhao et al. (2018)	China	VRMean: 59.38 ± 11.29 CGMean: 54.33 ± 10.93	24/24	Spastic	Xbox 360 Kinect	40m in/d^*^5 d/wk^*^3 wk	GMFM

E, experimental group; C, control group.

### 3.3 Quality assessment of the included studies

Table 2 presents the results of the methodology quality assessment of the included studies. A total of 24 RCTs were included in this study, with methodological quality scores ranging from 5 to 10 according to the PEDro scale; higher scores indicating a lower risk of bias. Of these, 20 RCTs were assessed as being at low risk of bias, and four were assessed as being at moderate risk. All 24 RCTs described the method of generating the random sequence, and six studies (Decavele et al., 2020; Pin and Butler, 2019; Fidan and Genç, 2023; El-Shamy and El-Banna, 2020; Bruno Arnoni et al., 2019; Jha et al., 2021) depicting a detailed description of the method of allocation concealment. Seven studies (El-Shamy and El-Banna, 2020; Bruno Arnoni et al., 2019; Kanitkar et al., 2023; Jha et al., 2021; Avcil et al., 2021; Chiu et al., 2014; Choi et al., 2021) implemented blinding of subjects, and a further five studies (Pin and Butler, 2019; El-Shamy and El-Banna, 2020; Bruno Arnoni et al., 2019; Cho et al., 2016; Chiu et al., 2014) used blinding of raters. Most studies had low blinding scores due to the inherent difficulty of implementing blinding in such studies. All 24 RCTs provided complete data and were not selectively reported.

**Table 2:** Methodological quality assessment for included studies.

**References**	**A**	**B**	**C**	**D**	**E**	**F**	**G**	**H**	**I**	**J**	**K**	**Score**
AlSaif and Alsenany (2015)	Yes	Yes	No	Yes	No	No	No	No	Yes	Yes	Yes	6/11
Bruno Arnoni et al. (2019) (D)	Yes	Yes	Yes	Yes	Yes	No	Yes	Yes	Yes	Yes	Yes	10/11
Bruno Arnoni et al. (2019) (E)	Yes	Yes	Yes	Yes	Yes	No	Yes	Yes	Yes	Yes	Yes	10/11
Chen et al. (2013)	Yes	Yes	No	Yes	No	No	No	Yes	Yes	Yes	Yes	7/11
Cho et al. (2016) (D)	Yes	Yes	No	Yes	No	No	Yes	Yes	Yes	Yes	Yes	8/11
Cho et al. (2016) (E)	Yes	Yes	No	Yes	No	No	Yes	Yes	Yes	Yes	Yes	8/11
Decavele et al. (2020)	Yes	Yes	Yes	No	No	No	No	Yes	Yes	Yes	Yes	7/11
Jha et al. (2021)	Yes	Yes	Yes	Yes	Yes	No	No	Yes	Yes	Yes	Yes	9/11
Fidan and Genç (2023)	Yes	Yes	Yes	Yes	No	No	No	Yes	Yes	Yes	Yes	8/11
Pin and Butler (2019)	Yes	Yes	Yes	Yes	No	No	Yes	Yes	Yes	No	Yes	8/11
Zhao et al. (2018)	Yes	Yes	No	Yes	No	No	No	Yes	Yes	Yes	Yes	7/11
Acar et al. (2016)	Yes	Yes	No	Yes	No	No	No	Yes	Yes	Yes	Yes	7/11
Avcil et al. (2021)	Yes	Yes	No	Yes	Yes	No	No	Yes	Yes	Yes	Yes	8/11
Chang et al. (2020)	No	No	No	Yes	No	No	No	Yes	Yes	Yes	Yes	5/11
Chiu et al. (2014)	Yes	Yes	No	Yes	Yes	No	Yes	Yes	Yes	Yes	Yes	9/11
Sharan et al. (2012)	Yes	Yes	No	Yes	No	No	No	Yes	Yes	Yes	Yes	7/11
El-Shamy and El-Banna (2020)	Yes	Yes	Yes	Yes	Yes	No	Yes	Yes	Yes	Yes	Yes	10/11
Choi et al. (2021)	Yes	Yes	No	Yes	Yes	No	No	Yes	Yes	Yes	Yes	8/11
Kanitkar et al. (2023)	Yes	Yes	No	Yes	Yes	No	No	Yes	Yes	Yes	Yes	8/11
Ren et al. (2016)	Yes	Yes	No	Yes	No	No	No	Yes	Yes	Yes	Yes	7/11
Ren et al. (2016) (D)	Yes	Yes	No	Yes	No	No	No	Yes	Yes	Yes	Yes	7/11
Ren et al. (2016) (E)	Yes	Yes	No	Yes	No	No	No	Yes	Yes	Yes	Yes	7/11
Saussez et al. (2023) (D)	Yes	Yes	No	Yes	No	No	No	No	Yes	Yes	Yes	6/11
Saussez et al. (2023) (E)	Yes	Yes	No	Yes	No	No	No	No	Yes	Yes	Yes	6/11

A. eligibility criteria were specified; B. subjects were randomly allocated to groups; C. allocation was concealed; D. the groups were similar at baseline regarding the most important outcome indicators; E. there was blinding of all subjects; F. there was blinding of all therapists; G. there was blinding of all assessors; H. measures of at least one key outcome were obtained from more than 85% of the subjects initially allocated to groups; I. all subjects for whom outcome measures were available received the treatment or, where this was not the case, data for at least one key outcome was analyzed by “intention to treat”; J. the results of between-group statistical comparisons were reported for at least one key outcome; K. the study provided both point measures and measures of variability for at least one key outcome.

### 3.4 Meta-analysis

#### 3.4.1 The effect of virtual reality motor games on gross motor skills in children with cerebral palsy

**Figure 2:**
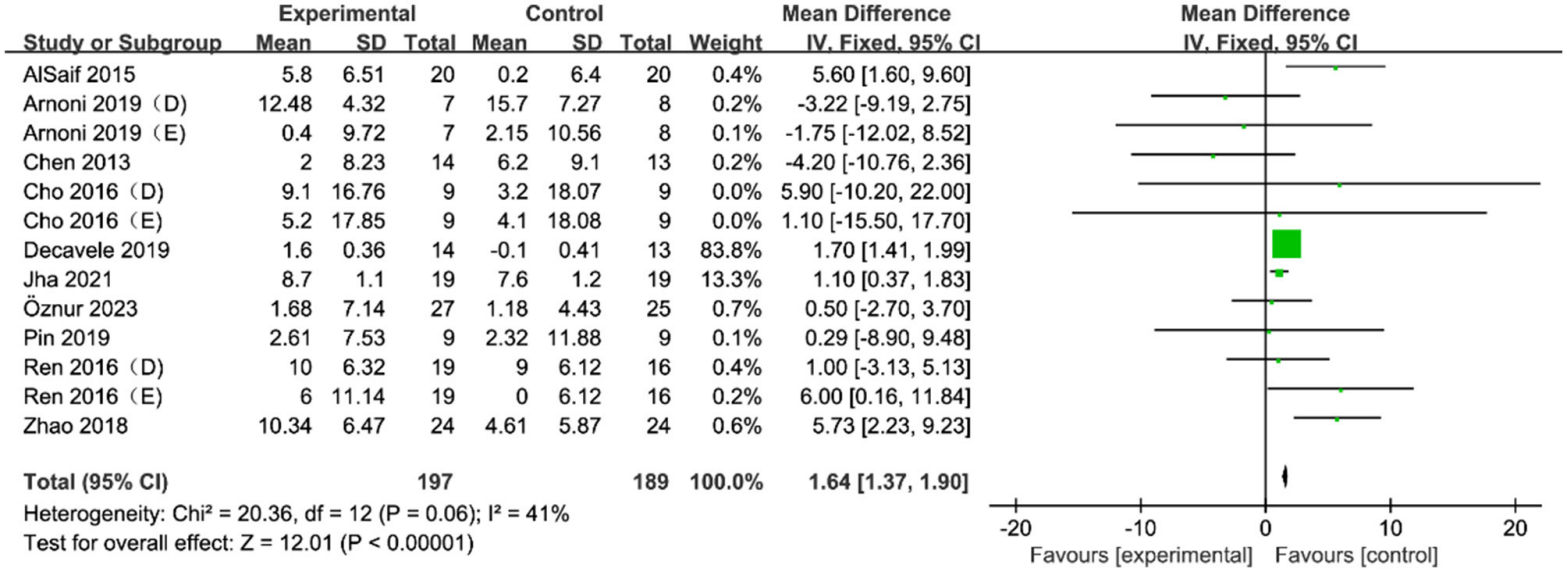
Forest plot of the effect of a VR motor game on gross motor skills in children with CP.

Thirteen RCTs involving 386 children with CP examined the effects of VR motor game training on their gross motor skills (Table 1). The studies assessed these skills using the MABC-2 and GMFM scales, as shown in Figure 2. The heterogeneity test results (*I*^2^ = 41%; *P* = 0.06) suggested minimal statistical heterogeneity among the studies, prompting the use of a fixed-effects model for the analysis. The meta-analysis demonstrated that VR motor game training significantly enhanced the gross motor skills of children with CP (MD = 1.67, 95% CI = 0.75–2.56; *P* < 0.001), indicating that VR training was more effective than the control group in improving gross motor skills in these children. As presented in the Table 3, the subgroup analysis categorized training frequency as ≤ 4 days per week and >4 days per week. It revealed that training more than 4 days per week significantly improved gross motor skills compared to the control group (*P* = 0.001). The training duration was divided into < 8 and ≥8 weeks, showing that both groups experienced significant improvements in gross motor skills compared to the control group (*P* < 0.00001 and *P* = 0.0007, respectively).

**Table 3:** Subgroup analysis of the effects of VR motor game training on gross motor skills in children with CP.

**Group**	**Study, n**	***I*^2^ (%)**	**Model**	**SMD (95% CI)**	***P-*value**
**Training frequency**					
≤ 4 days/week	6	18	Fixed effects model	1.60 (1.33, 1.87)	< 0.00001
>4 days/week	7	27	Fixed effects model	3.35 (1.63, 5.07)	=0.001
**Training cycle**					
< 8 weeks	5	41	Fixed effects model	1.72 (1.43, 2.01)	< 0.00001
≥8 weeks	8	26	Fixed effects model	1.19 (0.50, 1.88)	=0.0007

SMD, standardized mean difference.

#### 3.4.2 The effect of virtual reality motor games on fine motor skills in children with cerebral palsy

**Figure 3:**
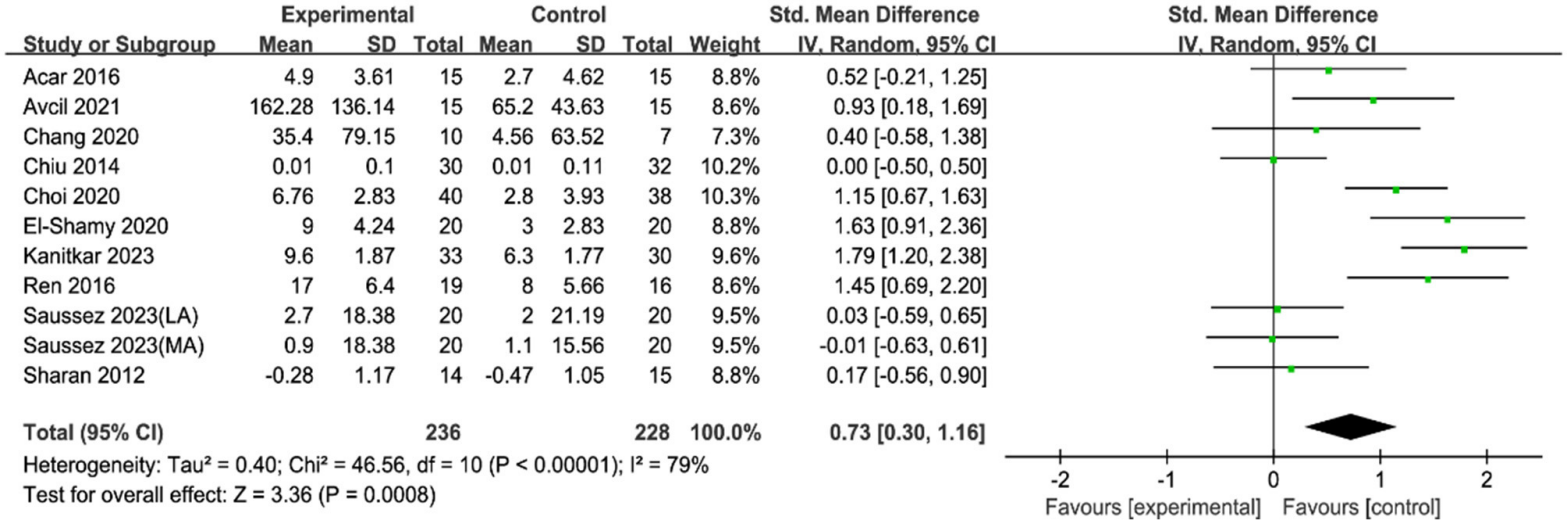
Forest plot of the effect of a VR motor game on fine motor skills in children with CP.

**Figure 4:**
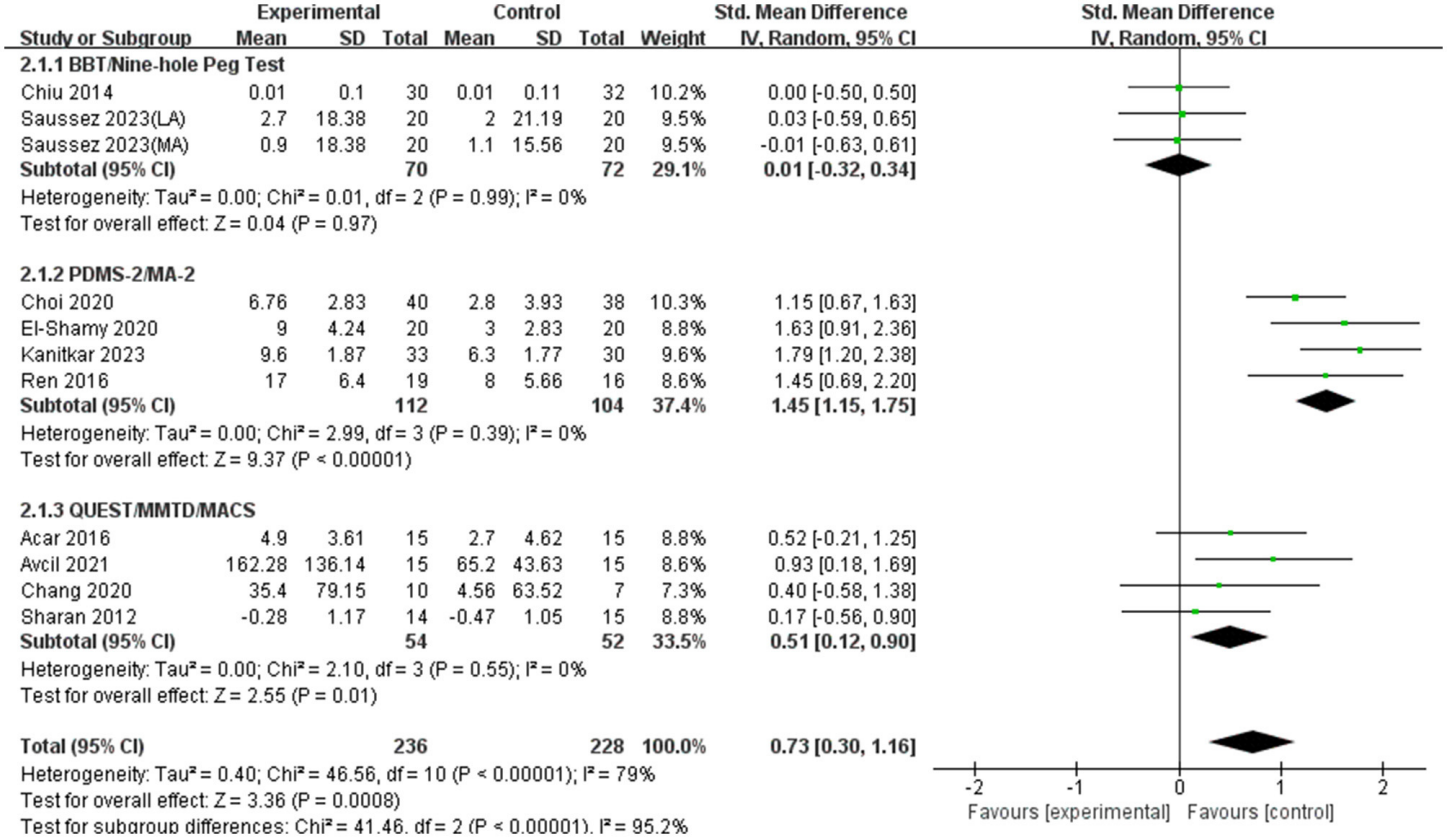
Subgroup analysis of the effects of VR motor game training on fine motor skills in children with CP.

Eleven RCTs involving 464 children with CP investigated the effects of VR motor game training on fine motor skills (Table 1). The results, as shown in the Figure 3, indicated significant statistical heterogeneity among the studies (*I*^2^ = 79%; *P* < 0.00001), prompting the use of a random-effects model for analysis. Subgroup analyses were conducted based on different scales to identify the source of this heterogeneity, revealing that the variability (*I*^2^ = 0) in each subgroup was due to the differences in measurement scales (Figure 4). The random-effects model analysis (SMD = 0.73, 95% CI = 0.30–1.16; *P* = 0.00008) showed that VR motor game training significantly improved fine motor skills in children with CP compared to the control group. The meta-analysis results confirmed that VR motor game training effectively enhanced the fine motor skills of these children (SMD = 0.73, 95% CI = 0.30–1.16; *P* = 0.00008) compared to conventional therapies.

#### 3.4.3 Testing of publication bias

Egger and Begg funnel plots were used in the study to test for publication bias in gross motor skills and fine motor skills. Using the Egger test, publication bias was detected as *Z* = 1.32 (Pr>|z| = 0.1874); gross motor skill was detected as *Z* = 1.52 (Pr>|z| = 0.1296); and fine motor skill was detected as *Z* = 0.9142 (Pr>|z| = 0.7555). The publication bias detection results using the Begg test were *Z* = −0.32 (Pr>|z| = 0.7850); gross motor skill detection result *Z* = −0.79 (Pr>|z| = 0.5022); and fine motor skill detection result Z = 0.7555 (Pr>|z| = 0.9142). Therefore, there was no publication bias in this study.

## 4 Discussion

### 4.1 Principal findings

VR motor games not only offer enhanced immersion but also provide an immersive and interactive environment that can significantly enhance the engagement and motivation of children with CP during therapy (Choi et al., 2021; Chen et al., 2018). This interactive setting is particularly beneficial as it allows children to be more involved and interested in their treatment, which is crucial for the success of any therapeutic intervention (Ravi et al., 2017). This is a critical factor, as traditional therapies can often be tedious and less engaging for children (Ashwini et al., 2021). A systematic review by Feitosa et al. (2022) supports this, highlighting that VR rehabilitation induces functional improvement and brain changes that can be detected by fMRI, indicating neuroplasticity resulting from these treatments. Moreover, VR interventions have been shown to be equally or more efficacious than traditional treatment methods in a majority of the studies reviewed, suggesting that VR provides a promising alternative to traditional therapies.

The primary goal of this study was to thoroughly assess the effects of VR motor games compared to other therapies on gross and fine motor skills in children with CP. The analysis included 24 RCTs, all involving children diagnosed with CP. The MABC-2 and GMFM scales were used to evaluate the gross motor skills of these children. The methodological quality assessment revealed a 41% likelihood of bias, and the meta-analysis indicated a combined effect size was 1.67. This result suggests that VR motor games significantly improved gross motor skills in children with CP compared to other therapies. This finding was confirmed by a meta-analysis by Ren and Wu (2019), which systematically evaluated the rehabilitation effects of VR games on gross motor skills and found that VR games could improve these skills. Furthermore, a study by Wu et al. (2019) supports our results, showing the rehabilitative effects of VR games on balance performance among children with CP, which is closely related to gross motor skills. These studies collectively suggest that VR motor games are an effective therapeutic intervention for improving gross motor skills in children with CP, reinforcing the potential of VR as a valuable tool in pediatric rehabilitation.

Fine motor coordination is crucial for daily activities such as eating, drinking, personal care, and manipulating small objects. It is particularly important for school-aged children, who frequently engage in tasks requiring high levels of hand-eye coordination, like coloring and writing (Johansson et al., 2001; Piek et al., 2006; Kaiser et al., 2009). The study results showed significant heterogeneity among the included studies. To identify the sources of heterogeneity, a subgroup analysis was conducted, dividing the data into three groups based on the assessment scales used. Each subgroup exhibited no statistical heterogeneity, indicating that the variation in assessment scales was the primary source of heterogeneity. The combined effect size for fine motor skills was 0.73, demonstrating that VR motor game training significantly and positively affected the fine motor skills of children with CP. This outcome is supported by a systematic review and meta-analysis by Kilcioglu et al. (2023), which investigated the short- to long-term effects of therapies including VR on motor skill learning in children with CP. The review found a significant short-term effect of adding VR to conventional therapies on upper limb functions, which are closely related to fine motor skills (Kilcioglu et al., 2023). A systematic review by Abdelhaleem et al. (2022) concluded that VR seems to be effective for improving fine motor coordination, with a large effect size observed. A study by Alharbi et al. (2024) also indicated that VR interventions appear promising in children and adolescents, suggesting potential benefits in motor skill enhancement and rehabilitation, including fine motor skills.

The findings of this review confirm that VR motor games are an effective intervention for improving the gross and fine motor abilities of children with CP, which is consistent with previous studies. These findings provide a scientific foundation for the clinical application of VR in pediatric rehabilitation, and VR motor games play an important role in pediatric rehabilitation.

### 4.2 Limitations

While this study offers some evidence for the benefits of VR motor games in improving motor skills in children with CP, several limitations exist. The study included a limited number of RCTs, which may affect the generalizability and applicability of the results. Moreover, since the VR intervention protocols and evaluation criteria were different for each of the studies, the comparability of findings was restricted. Even though a number of studies have shown that VR has a major effect on motor skills, more research is needed to determine the particular effects of VR on fine motor talents. A lack of long-term follow-up data is another issue with the majority of the studies that are currently in use, which leaves us with little knowledge of how long-term VR therapies will affect the long-term viability of these interventions. More high-quality, standardized RCTs should be used in future studies to corroborate these results and investigate the long-term consequences of various intervention conditions.

## 5 Conclusions

The findings from this systematic review and meta-analysis indicate that virtual reality motor games significantly enhance gross and fine motor skills in children with cerebral palsy. Among the 24 included RCTs, most studies demonstrated that virtual reality interventions improved motor skills more effectively than conventional rehabilitation training, primarily when conducted more than four times per week and lasting longer than 8 weeks. Nonetheless, some debate remains about the impact of virtual reality on fine motor skills in children with cerebral palsy. These results should be interpreted cautiously due to the limitations of the study methodology and the available studies.

## Data Availability

The original contributions presented in the study are included in the article/Supplementary material, further inquiries can be directed to the corresponding authors.
