# Supplementary material for: Effects of virtual reality motor games on motor skills in children with cerebral palsy: a systematic review and meta-analysis
Source: Front Psychol. 2025 Jan 6;15:1483370. doi: 10.3389/fpsyg.2024.1483370 (PMC11776641; doi:10.3389/fpsyg.2024.1483370)
Supplement: Supplementary file 1 [file Data_Sheet_1.zip › supplementary material/Table C Basic Features of the Included Studies.docx]

Table C: Basic Features of the Included Studies

| Author/Year | Country | Age (years) | Sample size（E/C） | CP Type | VR Intervention | Dosage | Outcome Indicators |  |
| --- | --- | --- | --- | --- | --- | --- | --- | --- |
|  |  |  |  |  |  |  |  |  |
| Acar 2016 | Istanbul | Range 6-15 | 15/15 | Spastic Diplegia | Nintendo Wii | 15min/d*2d/wk*6wk | QUEST |  |
| Avcil 2021 | Istanbul | Mean:10.0 ± 3.0 | 15/15 | Not specified | Nintendo Wii and LMC Games | 60min/d*3d/wk*8wk | MMDT |  |
| Chang 2020 | South Korea | VR:6.08 ± 1.77 CG:4.88 ± 1.15 | 10/7 | Not specified | RAPAEL Smart Kids | 20min/d*2d/wk*8wk | QUEST |  |
| Chiu 2014 | China-Taiwan | Range 6-13 | 32/30 | Spastic Diplegia | Wii Sports ResortTM | 40min/d*3d/wk*6wk | Nine-hole Peg Test |  |
| Choi 2020 | South Korea | Mean: 5.7±2.8 | 40/38 | Not specified | RAPAEL Smart Kids | 60min/d*5d/wk*4wk | MA-2 |  |
| El-Shamy 2020 | Saudi Arabia | Range8-12 | 20/20 | Spastic Diplegia | Nintendo Wii | 40 min/d*3d/wk*12wk | PDMS-2 |  |
| Kanitkar 2023 | Canada | Range 4-10 | 33/30 | Not specified | GRP | 45min/d*3d/wk*16wk | PDMS-2 |  |
| Saussez 2023 | Belgium | Range 5-18 | 20/20 | Spastic Diplegia | REAtouch | Not specified | BBT |  |
| Sharan 2012 | India | VR:8.88±3.23  CG:10.38±4.41 | 14/15 | Not specified | Nintendo Wii fit game | -min/d*2d/wk*3wk | MACS |  |
| AlSaif 2015 | Saudi Arabia | Range 6-10 | 20/20 | Spastic Diplegia | Nintendo Wii fit game | 20 min/d*7 d/wk*12 wk | mABC-2 |  |
| Arnoni 2019 | Brazilian | Mean: 10±3 | 7/8 | Not specified | Xbox 360 KinectTMand Kinect sensor | 45 min/d*2d/wk* 8wk | GMFM-88 |  |
| Chen 2013 | China | Range 6-12 | 14/13 | Spastic | Eloton SimCycle | 40 mim/d* 3 d/wk* 12 wk | GMFM-66 |  |
| Cho 2016 | South Korea | VR:10.2±3.4 CG:9.4±3.8 | 9/9 | Spastic | Nintendo Wii jogging program | 30min/d * 3 d/wk* 8 wk | GMFM |  |
| Decavele 2019 | Belgium | Range 6-15 | 14/13 | bilateral spastic | MS Kinect for Windows and Nintendo Wii balance board | 45 min/d*2 d/wk*12 wk | GMFM |  |
| Jha 2021 | India | Range 6-12 | 19/19 | bilateral spastic | Kinect-based virtual reality gaming | 60min/d*4d/wk*6wk | GMFM-88 |  |
| Öznur 2023 | Istanbul | MeanVR:9.2  MeanCG:9.4 | 27/25 | Spastic | XBox One Kinect | 45 min/d*2d/8wk*-wk | GMFM-88 |  |
| Pin 2019 | China-Hong Kong | EG:8.92±2.25 CG:9.59±1.87 | 9/9 | Not specified | Interactive computer play | 20min/d*4d/wk*6wk | GMFM-66 |  |
| Ren 2016 | China | Mean55.3±11.5 | 19/16 | bilateral spastic | Q4 Situational Interactive Training System | 40 min/d*5d/wk*12 wk | GMFM-88/PDMS-2 |  |
| Zhao 2018 | China | VRMean：59.38±11.29 CGMean：54.33±10.93 | 24/24 | Spastic | Xbox 360 Kinect | 40min/d*5d/wk*3wk | GMFM |  |
| E: Experimental Group; | |  |  |  |  |  |  |  |
| C：Control group; | |  |  |  |  |  |  |  |
