# Supplementary material for: Effects of virtual reality motor games on motor skills in children with cerebral palsy: a systematic review and meta-analysis
Source: Front Psychol. 2025 Jan 6;15:1483370. doi: 10.3389/fpsyg.2024.1483370 (PMC11776641; doi:10.3389/fpsyg.2024.1483370)
Supplement: Supplementary file 1 [file Data_Sheet_1.zip › supplementary material/Table B. Methodological Quality Assessment for Included Studies..docx]

Table B. Methodological Quality Assessment for Included Studies.

| Author(year) | A | B | C | D | E | F | G | H | I | G | J | score |
| --- | --- | --- | --- | --- | --- | --- | --- | --- | --- | --- | --- | --- |
| AlSaif 2015 | yes | yes | no | yes | no | no | no | no | yes | yes | yes | 6/11 |
| Arnoni 2019(D) | yes | yes | yes | yes | yes | no | yes | yes | yes | yes | yes | 10/11 |
| Arnoni 2019(E) | yes | yes | yes | yes | yes | no | yes | yes | yes | yes | yes | 10/11 |
| Chen 2013 | yes | yes | no | yes | no | no | no | yes | yes | yes | yes | 7/11 |
| Cho 2016(D) | yes | yes | no | yes | no | no | yes | yes | yes | yes | yes | 8/11 |
| Cho 2016(E) | yes | yes | no | yes | no | no | yes | yes | yes | yes | yes | 8/11 |
| Decavele 2019 | yes | yes | yes | no | no | no | no | yes | yes | yes | yes | 7/11 |
| Jha 2021 | yes | yes | yes | yes | yes | no | no | yes | yes | yes | yes | 9/11 |
| Öznur 2023 | yes | yes | yes | yes | no | no | no | yes | yes | yes | yes | 8/11 |
| Pin 2019 | yes | yes | yes | yes | no | no | yes | yes | yes | no | yes | 8/11 |
| Zhao 2018 | yes | yes | no | yes | no | no | no | yes | yes | yes | yes | 7/11 |
| Acar 2016 | yes | yes | no | yes | no | no | no | yes | yes | yes | yes | 7/11 |
| Avcil 2021 | yes | yes | no | yes | yes | no | no | yes | yes | yes | yes | 8/11 |
| Chang 2020 | no | no | no | yes | no | no | no | yes | yes | yes | yes | 5/11 |
| Chiu 2014 | yes | yes | no | yes | yes | no | yes | yes | yes | yes | yes | 9/11 |
| Sharan 2012 | yes | yes | no | yes | no | no | no | yes | yes | yes | yes | 7/11 |
| El-Shamy 2020 | yes | yes | yes | yes | yes | no | yes | yes | yes | yes | yes | 10/11 |
| Choi 2020 | yes | yes | no | yes | yes | no | no | yes | yes | yes | yes | 8/11 |
| Kanitkar 2023 | yes | yes | no | yes | yes | no | no | yes | yes | yes | yes | 8/11 |
| Ren 2016 | yes | yes | no | yes | no | no | no | yes | yes | yes | yes | 7/11 |
| Ren 2016(D) | yes | yes | no | yes | no | no | no | yes | yes | yes | yes | 7/11 |
| Ren 2016(E) | yes | yes | no | yes | no | no | no | yes | yes | yes | yes | 7/11 |
| Saussez 2023(D) | yes | yes | no | yes | no | no | no | no | yes | yes | yes | 6/11 |
| Saussez 2023(E) | yes | yes | no | yes | no | no | no | no | yes | yes | yes | 6/11 |

Note: a. eligibility criteria were specified; b. subjects were randomly allocated to groups; c. allocation was concealed; d. the groups were similar at baseline regarding the most important outcome indicators; e. there was blinding of all subjects; f. there was blinding of all therapists; g. there was blinding of all assessors; h. measures of at least one key outcome were obtained from more than 85% of the subjects initially allocated to groups; i. all subjects for whom outcome measures were available received the treatment or, where this was not the case, data for at least one key outcome was analyzed by “intention to treat”; j. the results of between-group statistical comparisons were reported for at least one key outcome; k. the study provided both point measures and measures of variability for at least one key outcome.
