# Supplementary figures and images for: Effects of virtual reality motor games on motor skills in children with cerebral palsy: a systematic review and meta-analysis
Source: Front Psychol. 2025 Jan 6;15:1483370. doi: 10.3389/fpsyg.2024.1483370 (PMC11776641; doi:10.3389/fpsyg.2024.1483370)

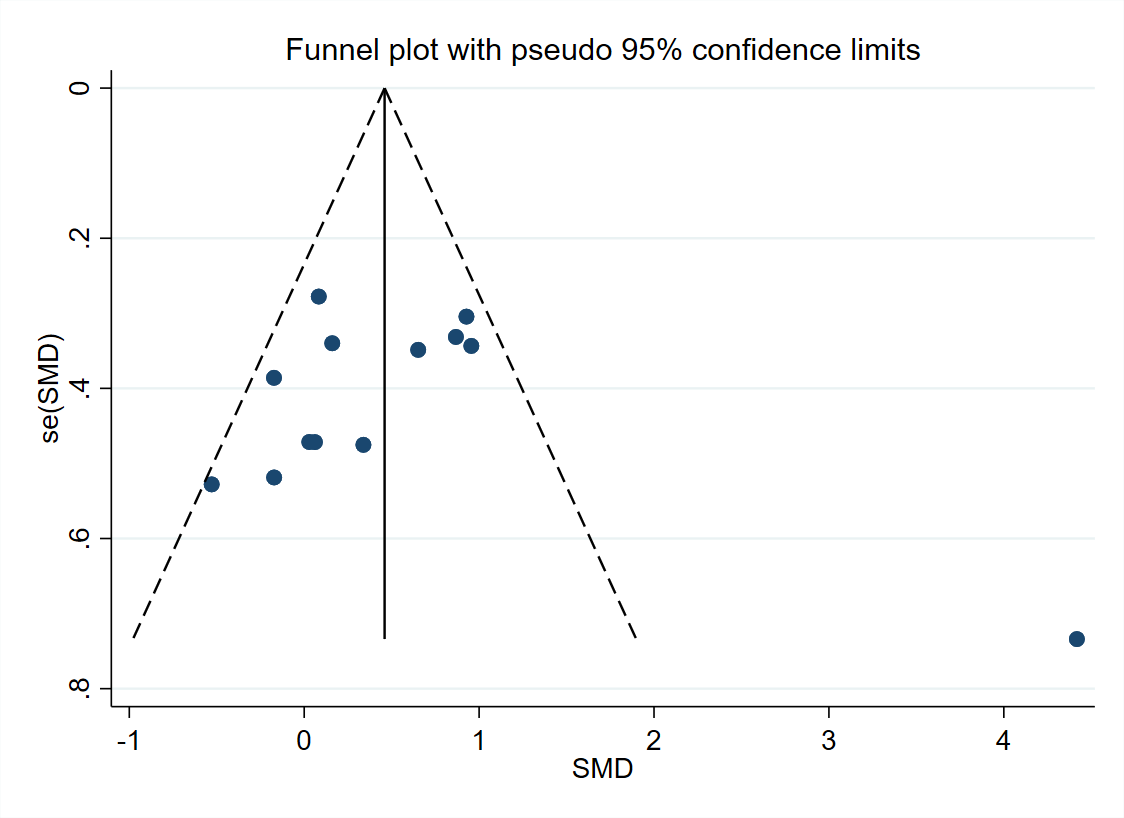

Supplement: Supplementary file 1 [file Data_Sheet_1.zip › supplementary material/Appendix D1 – Coarse Funnel plot.tif]

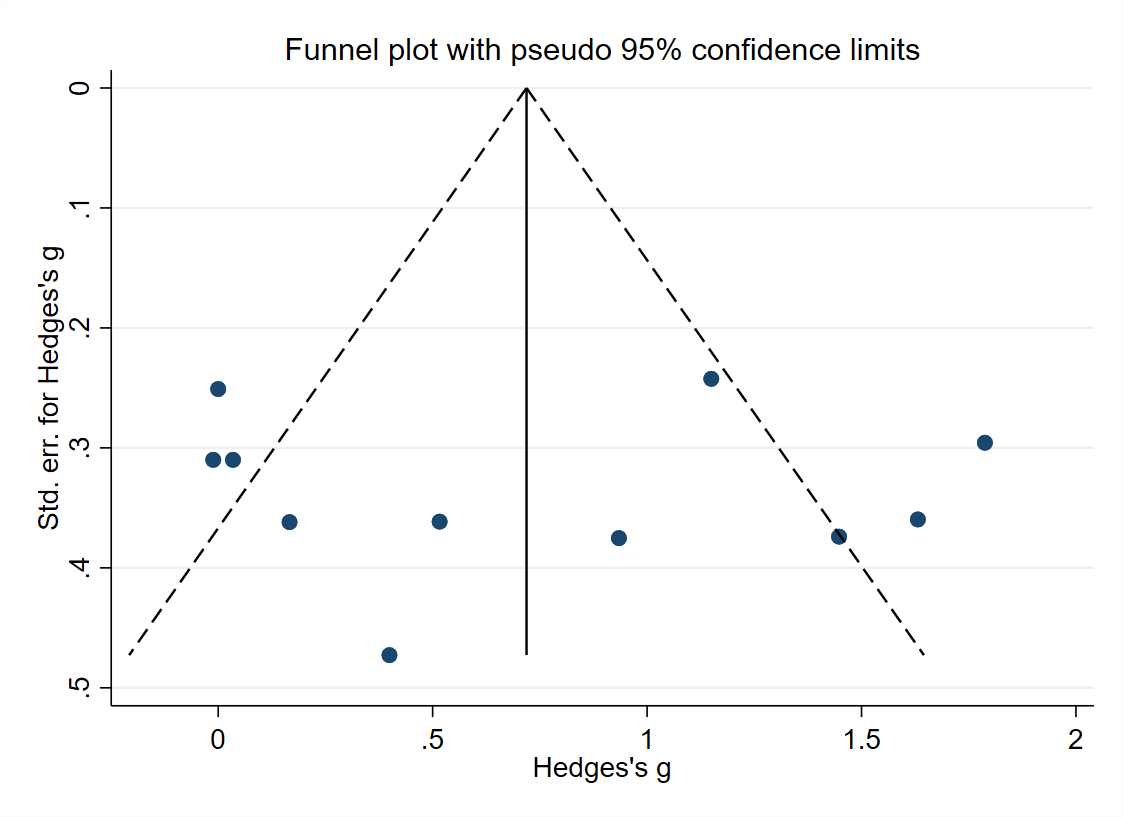

Supplement: Supplementary file 1 [file Data_Sheet_1.zip › supplementary material/Appendix D2 – Fine Funnel plot.tif]

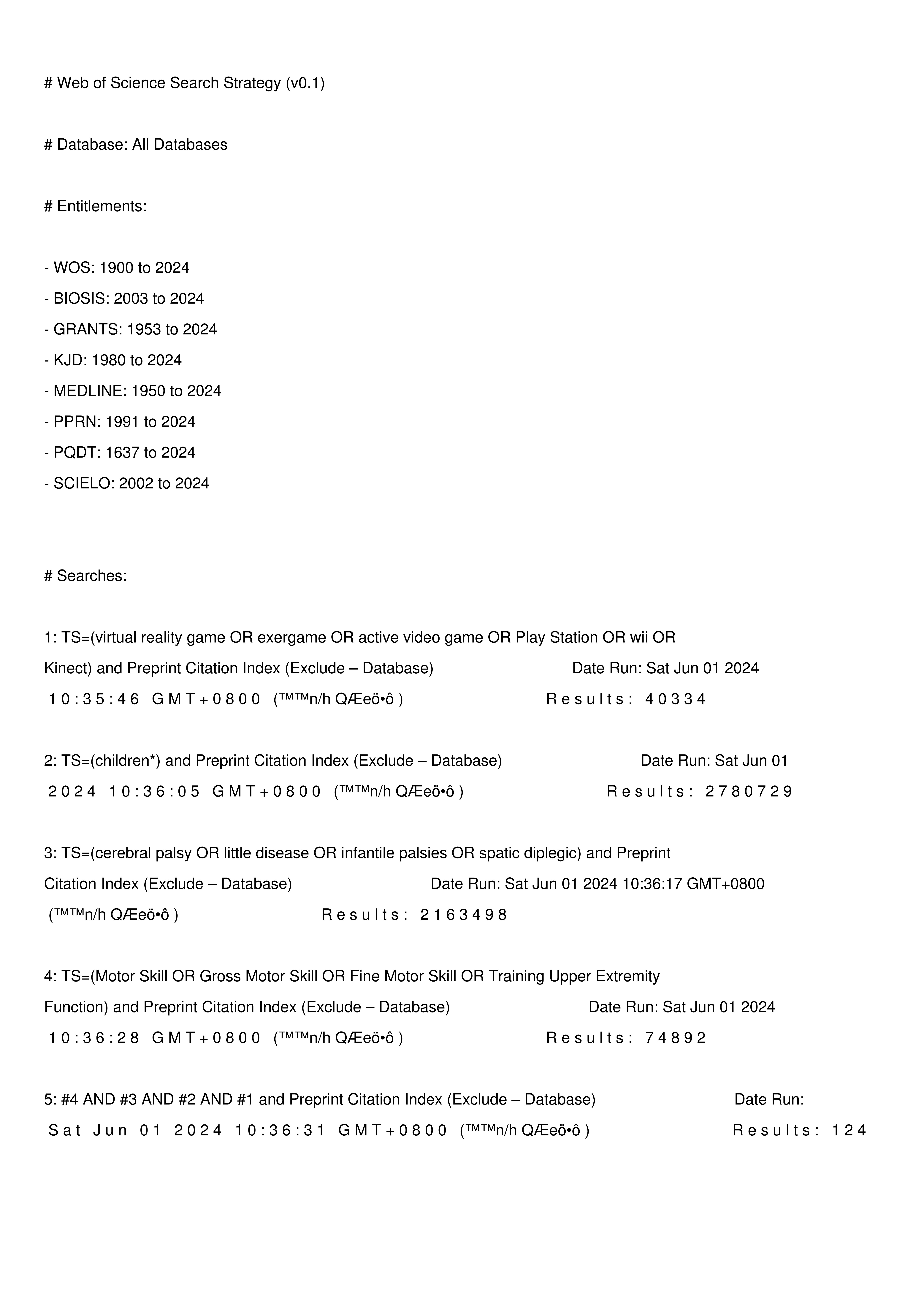

Supplement: Supplementary file 1 [file Data_Sheet_1.zip › supplementary material/Appendix A – Literature search terms.tif]
